# Trends in general and abdominal obesity in US adults: Evidence from the National Health and Nutrition Examination Survey (2001–2018)

**DOI:** 10.3389/fpubh.2022.925293

**Published:** 2022-10-06

**Authors:** Jin-Yu Sun, Wen-Jun Huang, Yang Hua, Qiang Qu, Chen Cheng, Heng-Li Liu, Xiang-Qing Kong, Yong-Xiang Ma, Wei Sun

**Affiliations:** ^1^Department of Cardiology, The Affiliated Jiangsu Shengze Hospital of Nanjing Medical University, Suzhou, China; ^2^Department of Cardiology, The First Affiliated Hospital of Nanjing Medical University, Nanjing, China; ^3^Geriatric Hospital of Nanjing Medical University, Nanjing, China

**Keywords:** waist circumference, body mass index, obesity, abdominal obesity, prevalence trend

## Abstract

**Aim:**

This study investigates the trend in general obesity and abdominal obesity in US adults from 2001 to 2018.

**Methods:**

We included 44,184 adults from the nine cycles of the continuous NHANES (2001–2002, 2003–2004, 2005–2006, 2007–2008, 2009–2010, 2011–2012, 2013–2014, 2015–2016, and 2017–2018). The age-adjusted mean body mass index and waist circumference were calculated, and the sex-specific annual change was estimated by the survey cycle. We used the weighted sex-specific logistic regression models to analyze the prevalence of general obesity and abdominal obesity from 2001 to 2018. The weighted adjusted odds ratio (OR) with a 95% confidence interval (CI) was calculated.

**Results:**

Our study showed that general obesity and abdominal obesity account for about 35.48 and 53.13% of the US population. From 2001–2002 to 2017–2018, the age-adjusted prevalence of general obesity increased from 33.09 to 41.36% in females and from 26.88 to 42.43% in males. During 2001–2018, the age-adjusted prevalence of abdominal obesity increased from 57.58 to 67.33% in females and from 39.07 to 49.73% in males. A significant time-dependent increase was observed in the prevalence of general obesity (adjusted OR, 1.007; 95% CI 1.005–1.009, *P* < 0.001) and abdominal obesity (adjusted OR, 1.006; 95% CI, 1.004–1.008; *P* < 0.001).

**Conclusion:**

General obesity and abdominal obesity are a heavy health burden among US adults, and the increasing trend remains in both males and females from 2001 to 2018.

## Introduction

Obesity refers to abnormal or excessive adiposity accumulation. As a common, complex, and costly disease, obesity has become a significant threat to socio-economic stability and public health worldwide over the past few decades (1, 2). In a pooled analysis of 128.9 million children, adolescents, and adults, the obesity prevalence was reported to increase in every country between 1975 and 2016 (3). According to World Health Organization, obesity has nearly tripled worldwide since 1975. In 2016, more than 1.9 billion (39%) adults were overweight, and over 650 million (13%) were obese (4).

Body mass index (BMI) is the most widely used anthropometric indicator to define obesity. Studies have demonstrated high BMI as an independent risk factor for multiple diseases, such as cardiovascular diseases, diabetes mellitus, and cancer (5–8). However, BMI provides only a crude measure of general obesity and fails to fully capture the heterogeneous regional body fat distribution. Concerns were raised on the limitation of BMI in evaluating the individual-level metabolic risk (9). Compared with BMI, waist circumference is strongly associated with abdominal fat distribution and is a better index for abdominal obesity. The combination of BMI and waist circumference could provide additional opportunities to prevent obesity-related adverse events (10–13). Individuals with low BMI but abdominal obesity are at higher risk for multiple diseases and all-cause mortality (14, 15).

Considering the substantial obesity burden, the obesity prevalence has been continuously surveilled, and an updated investigation of obesity prevalence is necessary. Several studies have reported the prevalence trend of obesity in the United States and many other countries (16–18). The overall prevalence trend of obesity in US adults has been previously described using national survey data, and a significantly increased prevalence of BMI-defined obesity was observed from the 1970s−2000s (19–21). Liu et al. (22) examined the trends in body mass index, waist circumference, and body fat percentage from 2011 to 2018. Similarly, Flegal et al. (23) reported the obesity trends in US adults from 2005 to 2014, but a broad overview on the prevalence trend of obesity during the first two decades of the 21st century is lacking. Recent studies suggested that the combination of BMI and waist circumference could provide an additional opportunity for the primary prevention of cardiovascular diseases (24–29). However, few studies reported the obesity prevalence trend based on the combination of BMI and waist circumference (e.g., normal-weight abdominal obesity).

This study analyzed the National Health and Nutrition Examination Survey (NHANES) from the first continuous survey in the 2lst century to the last survey before the COVID-19 pandemic (30). We described the prevalence of (abdominal) obesity and dynamic change in BMI and waist circumference during 2001–2018. We also analyzed obesity trends based on the combination of BMI and waist circumference. This study provides a comprehensive insight into how general obesity and abdominal obesity have developed in the United States over the past 18 years.

## Methods

### Data source

National Health and Nutrition Examination Survey (NHANES) is a continuous cross-sectional survey by the National Center for Health Statistics in the Centers for Disease Control and Prevention, which records US populations' health and nutrition information. The continuous NHANES has been conducted once every 2 years since 1999. A complex stratified multistage-clustered sampling design was applied to select the nationally representative participants. In this study, we obtained adults from the nine cycles of the continuous NHANES (2001–2002, 2003–2004, 2005–2006, 2007–2008, 2009–2010, 2011–2012, 2013–2014, 2015–2016, and 2017–2018). The exclusion criteria were as follows: (1) missing weight, height, or waist circumference records, (2) aged ≥80 years old, and (3) pregnant individuals. Finally, 44,184 individuals were enrolled in this study, including 22,075 females and 22,109 males. NHANES was approved by National Center for Health Statistics Research Ethics Review Board, and informed consent was obtained from all participants (31).

### Body mass index and waist circumference measurement

Weight and height were measured by trained examiners using standardized techniques and equipment. BMI was calculated by the following formula: BMI = $\frac{weight\;\left(kg\right)}{{height\;\left(m\right)}^{2}}$; values were rounded to the nearest 0.1 kg/m^2^ for presentation purposes. Based on the World Health Organization criterion, BMI was later transferred to a categorical variable: underweight (<18.5 kg/m^2^), normal weight (18.5–24.9 kg/m^2^), overweight (25.0–29.9 kg/m^2^), class I obese (30.0–34.9 kg/m^2^), class II obese (35.0–39.9 kg/m^2^), and class III obese (≥40.0 kg/m^2^).

Waist circumference was measured around the waist at the level of the superior lateral border of the iliac crests. A trained examiner recorded waist circumference to the nearest 0.1 cm after the participant exhaled one normal breath. Males with waist circumference ≥100.0 cm and females with ≥88.0 cm were classified as having abdominal obesity (32). In addition, we classified the individuals into four subgroups according to the combination of BMI and waist circumference: BMI < 30.0 with abdominal obesity; BMI < 30.0 without abdominal obesity; BMI ≥ 30.0 with abdominal obesity; and BMI ≥ 30.0 without abdominal obesity.

### Statistical analysis

We performed data analyses according to the recommendation by the NHANES analytic and reporting guidance document (33). NHANES applied a complex survey design to reduce the bias caused by oversampling, non-response, and post-stratification. Each participant was assigned a specific sampling weight, primary sampling unit (PSU), and stratum, which could be used to produce representative national estimates. WTMEC2YR records the survey weight for all participants who received body measures in a Mobile Exam Center. Since we analyzed nine cycles of the continuous NHANES, we calculated the 18-years sample weight (WTMEC18YR) by the following formula: $WTMEC18YR=\;\frac{1}{9}\times WTMEC2YR$. We applied survey weights in subsequent statistical analysis to produce estimates representative of the US population.

Continuous variables were represented as weighted mean ± standard error, and categorical variables were represented with proportions. Sex-specific distributions of BMI and waist circumference were illustrated using kernel density estimation. The sex-specific distribution of BMI and waist circumference was further shown in three intervals, including NHANES 2001–2006, 2007–2012, and 2013–2018.

We illustrated the mean BMI and waist circumference by survey cycle. Following a previous study (20), the time-related change was evaluated by a survey-weighted linear regression taking 2-years cycles as a continuous variable. Importantly, we used the weighted sex-specific logistic regression models to analyze the prevalence of general obesity and abdominal obesity. Age, race, and education levels were adjusted in this model. The weighted odds ratio (OR) with a 95% confidence interval (CI) was calculated. Moreover, we analyzed the association of age with BMI and waist circumference using a sex-specific survey-weighted generalized linear model in the young adults (<45 years old), middle-age adults (45–65 years old), and older adults (≥65 years old), respectively. All statistical analyses were performed in R software (version 4.1.1). *P*-value < 0.05 was considered statistically significant.

## Results

### Participant characteristics

**Figure 1:**
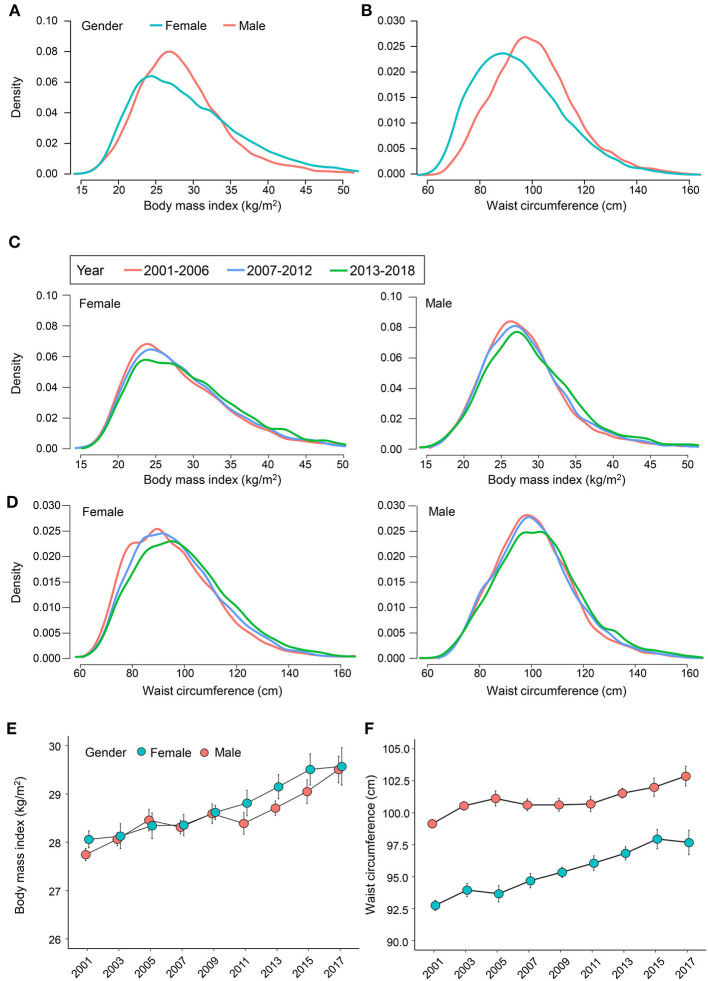
Distribution trend of body mass index and waist circumference during 2001–2018. The sex-specific distribution of **(A)** body mass index and **(B)** waist circumference. The sex-specific distribution of **(C)** body mass index and **(D)** waist circumference in 2001–2006, 2007–2012, and 2013–2018. The mean age-adjusted **(E)** body mass index and **(F)** waist circumference from 2001 to 2018. The weighted study design is considered in the analysis.

Table 1 summarizes the characteristics of all participants in this study. Overall, the mean age was 44.65 years with a standard error of 0.19. Race and ethnicity were non-Hispanic White (67.10%), followed by non-Hispanic Black (11.38%) and Mexican American (8.66%). Normal weight, overweight, and obese individuals account for about 30.04, 32.69, and 35.48% of the total population. In individuals with obesity, the percentage of class I obesity ranked first (20.17%), followed by class II obesity (8.98%) and class III obesity (6.32%). Abdominal obesity was observed in 53.13% of all populations, with a significantly higher prevalence in females (62.92 vs. 43.15%; *P* < 0.001). In those with combined obese classification, BMI < 30.0 kg/m^2^ without abdominal obesity was the primary subtype (45.50%), followed by BMI ≥ 30.0 kg/m^2^ with abdominal obesity (34.11%), BMI < 30.0 kg/m^2^ with abdominal obesity (19.02%), and BMI ≥ 30.0 kg/m^2^ without abdominal obesity (1.37%). Females showed significantly lower waist circumference (95.45 vs. 100.66 cm; mean difference, −5.20 cm; *P* < 0.001), whereas BMI (28.78 vs. 28.54 kg/m^2^; mean difference, 0.24 kg/m^2^; *P* = 0.005) was slightly higher in females than males. The sex-specific smoothed density distribution of BMI and waist circumference was displayed in Figures 1A,B. Abdominal obesity showed a higher prevalence in females in both subgroups with BMI < 30.0 kg/m^2^ and ≥30.0 kg/m^2^. For subjects with BMI < 30.0 kg/m^2^, 41.54% of females and 17.74% of males had abdominal obesity, while for those with BMI ≥ 30.0 kg/m^2^, the prevalence of abdominal obesity was 99.43 and 92.50%, respectively.

**Table 1:** Participant characteristics.

	**All**	**Female**	**Male**
*N*		22,075	22,109
Age (years)	44.652 ± 0.19	45.31 ± 0.2	43.98 ± 0.20
Education (*n*, %)			
Below high school	15.94	15.10	16.81
High school	23.75	22.64	24.90
Above high school	60.31	62.26	58.30
Race/ethnicity (*n*, %)			
Non-hispanic white	67.10	66.74	67.46
Non-hispanic black	11.38	12.13	10.61
Mexican American	8.66	7.97	9.36
Other hispanic	5.58	5.77	5.39
Other race	7.28	7.39	7.18
BMI (kg/m^2^)	28.78 ± 0.09	28.78 ± 0.09	28.54 ± 0.07
BMI (categories, %)			
Normal weight	30.04	33.24	26.79
Underweight	1.79	2.31	1.27
Overweight	32.69	27.51	37.95
Obese	35.48	36.94	33.99
Waist circumference (cm)	95.45 ± 0.22	95.45 ± 0.22	100.66 ± 0.21
Abdominal obesity (yes, %)	53.13	62.92	43.15
Combined obesity (%)			
BMI < 30 without abdominal obesity	45.50	36.87	54.30
BMI < 30 with abdominal obesity	19.02	26.19	11.71
BMI ≥ 30 without abdominal obesity	1.37	0.21	2.55
BMI ≥ 30 with abdominal obesity	34.11	36.73	31.44

Weighted analysis was performed. BMI, body mass index.

Figures 1C,D shows the sex-specific density curve of BMI and waist circumference by year intervals (2001–2006, 2007–2012, and 2013–2018). A rightward shift was observed in the distributions of BMI and waist circumference. The weighted mean BMI (β-coefficient, 0.10; *P* < 0.001) and waist circumference (β-coefficient, 0.28; *P* < 0.001) significantly increased over the 18-years survey. The age-adjusted mean BMI has increased from 28.06 to 29.57 kg/m^2^ in females and from 27.74 to 29.50 kg/m^2^ in males (Figure 1E), whereas the age-adjusted mean waist circumference increased from 92.75 to 97.67 cm in females and from 99.14 to 102.86 cm in males (Figure 1F). Interestingly, between 2015–2016 and 2017–2018, the increase in BMI showed a slowing trend (from 29.51 to 29.57 kg/m^2^) in females, and a decrease in waist circumference (from 97.94 to 97.67 cm) was also observed.

### The prevalence of general and abdominal obesity

**Figure 2:**
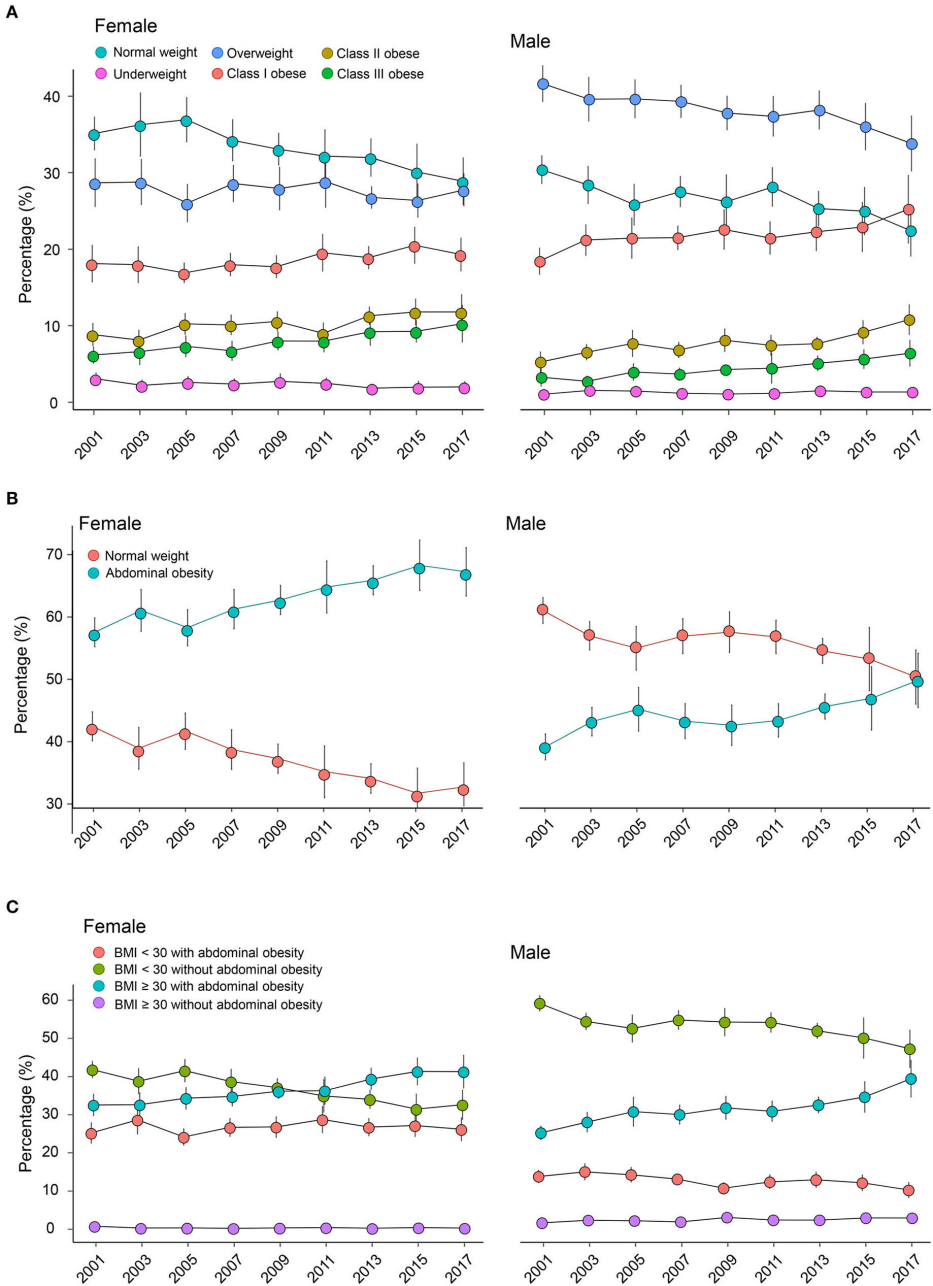
Weighted age-adjusted prevalence trend with a 95% confidence interval of **(A)** general obesity, **(B)** abdominal obesity, and **(C)** combined obesity during 2001–2018 in females and males. Obesity is classified based on the World Health Organization criterion: underweight (<18.5 kg/m^2^), normal weight (18.5–24.9 kg/m^2^), overweight (25–29.9 kg/m^2^), class I obese (30–34.9 kg/m^2^), class II obese (35–39.9 kg/m^2^), and class III obese (≥40 kg/m^2^). Females with waist circumference ≥88 cm and males with ≥100 cm are identified with abdominal obesity. The combined obesity is defined according to the combination of BMI and waist circumference: BMI < 30 with abdominal obesity; BMI < 30 without abdominal obesity; BMI ≥ 30 with abdominal obesity; BMI ≥ 30 without abdominal obesity. *BMI, body mass index*.

Table 2 shows significant association of survey year with general obesity (adjusted OR, 1.007; 95% CI, 1.005–1.009, *P* < 0.001) and abdominal obesity (adjusted OR, 1.006; 95% CI, 1.004–1.008; *P* < 0.001). From 2001 to 2018, we observed a decreasing trend in the normal-weight group (female, 35.16–28.90%; male, 30.42–22.43%), while the percentage of underweight remained stable. From 2001–2002 to 2017–2018, the age-adjusted prevalence of general obesity increased from 33.09 to 41.36% in females and from 26.88 to 42.43% in males. In females, the prevalence of overweight showed a fluctuating trend, whereas the percentage of class II (from 8.83 to 11.80%) and III obese (from 6.15 to 10.25%) showed an about 1.5-fold increase. In contrast, males showed a decreasing overweight trend, while the increased prevalence was observed in all three different classes of obese (class I, from 18.43 to 25.25%; class II, from 5.23 to 10.79%; and class III, from 3.22 to 6.39%; Figure 2A). In addition, we also provided the obesity prevalence based on the categorization of underweight, normal weight, overweight, obese (BMI, 30.0–39.9 kg/m^2^), and morbidly obese/severely obese (BMI ≥ 40 kg/m^2^) in Supplementary Figure 1. During 2001–2018, the age-adjusted prevalence of abdominal obesity increased from 57.58 to 67.33% in females and from 39.07 to 49.73% in males. Females and males showed a similar increasing trend in abdominal obesity, and the prevalence of abdominal obesity was consistently higher in females (Figure 2B). The prevalence of abdominal obesity in males has increased close to that of normal weight. Importantly, we have observed a reversed trend of abdominal obesity in females since 2015. Figure 2C describes the trend of the combined obesity based on BMI and waist circumference. The percentage of the group of BMI < 30.0 kg/m^2^ without abdominal obesity has been decreasing, while the group of BMI ≥ 30.0 kg/m^2^ with abdominal obesity showed a consistently increasing trend. In the subgroup of BMI ≥ 30.0 kg/m^2^, the percentage of those without abdominal obesity remained low, while those with abdominal obesity showed an increasing trend.

**Table 2:** Time-related change in the prevalence of obesity and abdominal obesity (weighted).

	**General obesity**			**Abdominal obesity**	
	**OR**	**95% CI**	***P***	**OR**	**95% CI**	***P***
Female						
Year	1.006	(1.004–1.009)	<0.001	1.008	(1.005–1.010)	<0.001
Survey						
2001–2002	*Reference*			*Reference*		
2003–2004	1.003	(0.959–1.049)	0.897	1.045	(0.998–1.093)	0.058
2005–2006	1.025	(0.983–1.068)	0.247	1.019	(0.978–1.061)	0.365
2007–2008	1.02	(0.982–1.059)	0.304	1.046	(1.005–1.09)	0.029
2009–2010	1.032	(0.998–1.067)	0.064	1.064	(1.027–1.102)	<0.001
2011–2012	1.045	(0.998–1.094)	0.064	1.095	(1.045–1.148)	<0.001
2013–2014	1.077	(1.035–1.121)	<0.001	1.108	(1.072–1.144)	<0.001
2015–2016	1.100	(1.055–1.146)	<0.001	1.135	(1.087–1.184)	<0.001
2017–2018	1.095	(1.040–1.153)	<0.001	1.117	(1.066–1.171)	<0.001
Male						
Year	1.008	(1.005–1.010)	<0.001	1.005	(1.003–1.008)	<0.001
Survey						
2001–2002	*Reference*			*Reference*		
2003–2004	1.037	(1.002–1.074)	0.040	1.043	(1.012–1.076)	0.007
2005–2006	1.065	(1.018–1.115)	0.007	1.061	(1.017–1.108)	0.007
2007–2008	1.051	(1.018–1.086)	0.003	1.049	(1.014–1.084)	0.006
2009–2010	1.086	(1.044–1.129)	<0.001	1.041	(1.005–1.079)	0.027
2011–2012	1.074	(1.038–1.110)	<0.001	1.056	(1.019–1.095)	0.003
2013–2014	1.085	(1.053–1.118)	<0.001	1.075	(1.046–1.105)	<0.001
2015–2016	1.117	(1.065–1.170)	<0.001	1.091	(1.035–1.151)	0.001
2017–2018	1.174	(1.110–1.241)	<0.001	1.125	(1.071–1.181)	<0.001

A weighted sex-specific logistic regression model was used to analyze the trend of general obesity and abdominal obesity. The prevalence in the 2001–2002 survey was set as the reference. Age, race, and education levels were adjusted for in the model. The weighted OR with a 95% CI was calculated. BMI, body mass index; OR, odds ratio; CI, confidence interval.

### The association of age, BMI, and abdominal obesity

**Figure 3:**
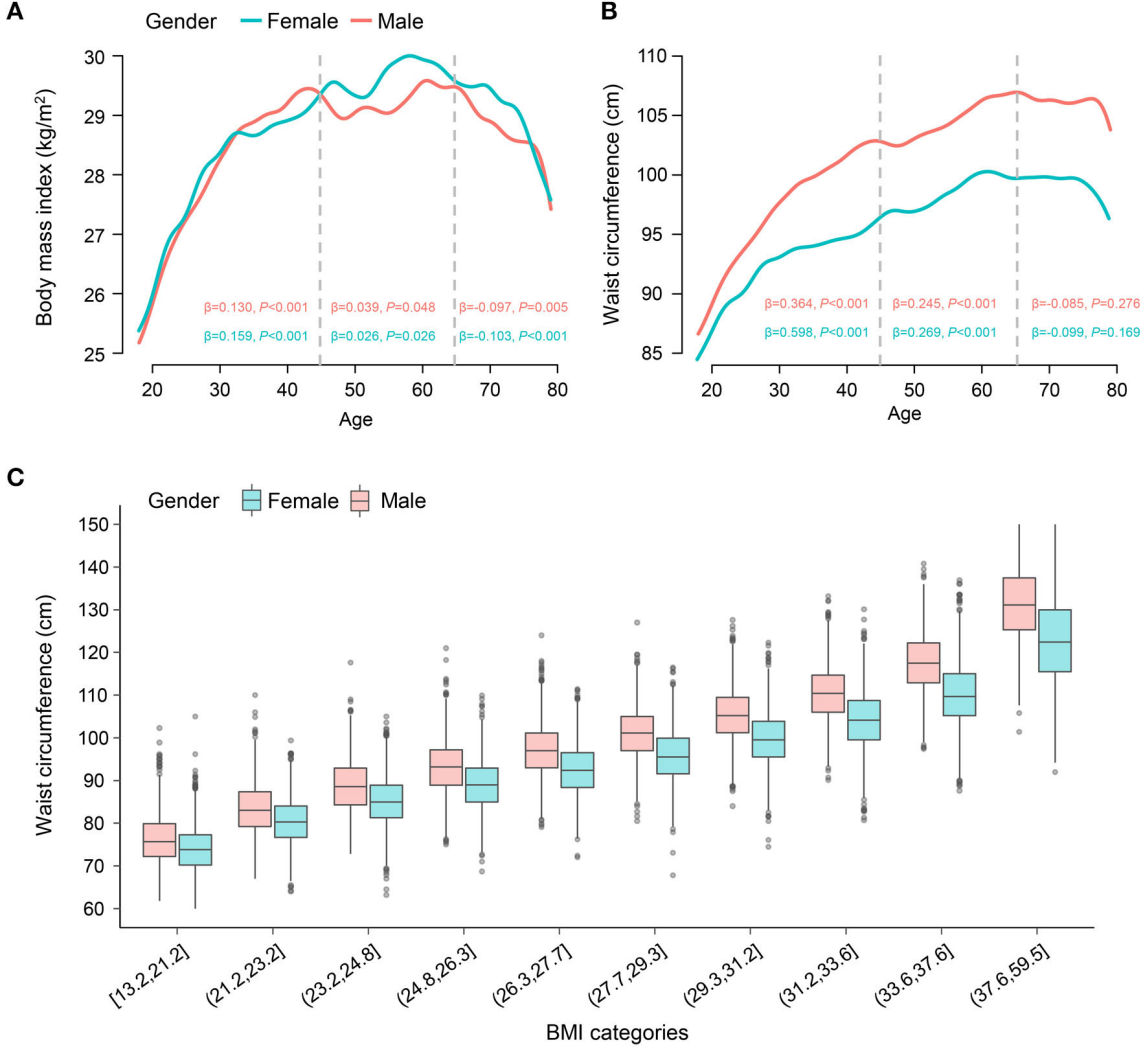
Association of age with BMI and waist circumference. The smoothed curve on the association of age with **(A)** BMI and **(B)** waist circumference. The association of age with BMI and waist circumference is analyzed by a sex-specific survey-weighted generalized linear model in the young adults (<45 years old), middle-age adults (45–65 years old), and older adults (≥65 years old) individuals, respectively. The β-coefficient with *P*-value is provided. **(C)** Boxplots on the distribution of waist circumference across BMI (ten categories) by gender. Weighted analysis was performed. *BMI, body mass index*.

Figures 3A,B shows smoothed curves of age with BMI and waist circumference. BMI and waist circumference significantly increased with aging in young adults. Alternatively, among middle-age adults, the curve was relatively flat in BMI, while the increasing trend remained in waist circumference. In the subgroup of older adults, the negative association was observed between aging and BMI (female, β-coefficient = −0.097, *P* = 0.005; male, β-coefficient = −0.103, *P* < 0.001). Differently, the curve for waist circumference remained stable after 65 years old, and no significant association was observed (*P* > 0.05).

Figure 3C uses boxplots to illustrate waist circumference distribution across BMI (ten categories) by gender. Waist circumference maintained sustainable growth with the increase in BMI. Still, although individuals in the same category shared similar BMI, their waist circumference showed a relatively large range.

## Discussion

Obesity is an independent risk factor for multiple chronic conditions, such as cardiovascular diseases, stroke, and cancers. Across regions and countries, obesity remains a significant public health problem with a varying prevalence (34). Using nationally representative samples, our study showed that individuals with overweight or obesity account for about 32.69 and 35.48% of the US population. Abdominal obesity was observed in 53.13% of all populations, with a significantly higher prevalence in females than males.

Considering the substantial obesity burden and the obesity-induced health risk in the United States, the obesity prevalence has been continuously surveilled (35). The prevalence of general obesity showed a flat trend from the 1960s until the 1980s (21). After that, the prevalence of obesity increased, and the distribution changed. A rapid increase was observed from 1976–1980 to 1988–1994 survey (8.9% in females and 7.9% in males; 21) and again between 1988–1994 and 1999–2000 (8.1% in females and 7.1% in males; 19–21). However, over the 1999–2008 period, the prevalence of obesity increased by 4.7% (95% CI, 0.5–9.0%) in males, and a nonsignificant increase was shown in 2.1% (95% CI, −2.1–6.3%) in females (36). Despite the significant linear trend in males, there was no significant difference between 2003–2004, 2005–2006, and 2007–2008. The study by Flegal et al. (36) suggested that the increasing trend of obesity seemed to slow down in the US population during 1999–2008, particularly for women and possibly for men. In the following research, Liu et al. (22) investigated the trends of body mass index and waist circumference from NHANES 2011–2018. Age-adjusted mean BMI significantly increased from 28.7 to 29.8 kg/m^2^ during 2011–2018, and age-adjusted mean waist circumference increased from 98.4 to 100.5 cm. The increase in the prevalence of obesity has also been observed in many other countries or areas, including China (18), Bissau (17), and Mozambique (16).

Compared with previous studies (16–18, 22, 23), we analyzed the obesity prevalence from the first continuous NHANES survey in the 2lst century (2001–2002 survey) to the last survey before the COVID-19 pandemic (2017–2018). The 18-years investigation provides a more comprehensive understanding of how general obesity and abdominal obesity develop in the United States. Importantly, the nationally representative data source with a stratified multistage-clustered sampling design allows the accurate evaluation of the US obesity prevalence. From 2001–2002 to 2017–2018, we observed a rightward shift in BMI distribution. In females, the prevalence of overweight fluctuated, while the percentage of class II and III obese increased by about 50%. Also, the logistic regression suggested no significant difference in the female obesity prevalence between 2001 and 2002 and the other surveys (2003–2004, 2005–2006, 2007–2008, 2009–2010, and 2011–2012). Still, a significant increase was observed from 2013 to 2014 with an adjusted OR of 1.077 (95% CI, 1.035–1.121; 2013–2014 vs. 2001–2002). In males, the prevalence of overweight exhibited a decreasing trend, but a significant increase was observed in all three classes of obesity.

BMI is the most widely used anthropometric index to diagnose obesity, providing a practical tool for national surveillance and international comparisons (37). However, abdominal adipose accumulation is a higher risky obesity phenotype than excess subcutaneous fat distribution (9). BMI fails to consider the body fat distribution and could not distinguish fat from lean mass (9, 38–40). Therefore, BMI-defined obesity is insufficient to fully capture the obesity-related metabolic risk. Previous studies have demonstrated that waist circumference is closely associated with multiple cardiovascular diseases and all-cause mortality independent of BMI (27, 41). When evaluating obesity-related metabolic risk, these data supported that waist circumference and BMI were irreplaceable adiposity indexes. However, waist circumference is not routinely measured in clinical practice, and fewer studies reported the prevalence of abdominal obesity based on waist circumference. Over the past 18 years, the waist circumference distribution has been rightward shifted. For abdominal obesity, we observed a consistently increasing trend in both males and females, and its prevalence among females has grown to almost 50% in the 2017–2018 survey. Also, we observed that waist circumference was parallel to BMI, while waist circumference ranges even in the same BMI category. Our data suggest that abdominal obesity remains a growing health problem in the United States, especially among males. Importantly, we also analyzed the trend based on the combination of BMI and waist circumference, which provides additional opportunities to prevent obesity-related adverse events.

The prevalence of obesity and abdominal obesity in females is another important topic. Our study showed that females were more likely to be obese or have abdominal obesity in the United States, consistent with most other countries (42). Interestingly, from 2015–2016 to 2017–2018, we observed a small change in the mean BMI (29.51–29.57 kg/m^2^) and a decreased trend in waist circumference (97.94–97.67 cm) in females. These data indicated a decrease in the prevalence of obesity in females. However, it is challenging to decide the future trends based on current information since we are unaware of the underlying causes.

Our study has several strengths and limitations. The main strength was the application of NHANES, one of the most authoritative and reliable resources for epidemiological health and nutritional survey in the United States. NHANES allowed us to assess the national trends of obesity by documenting demographic information, including race/ethnicity, height, weight, and waist circumference measured by trained investigators. Still, some limitations in our study should be noted. First, although NHANES applied a complex survey design, sampling or the non-sampling error could not be entirely avoided. Second, the prevalence of obesity varies across regions and countries, and obesity in developing countries has become a growing problem within decades due to rapid social and economic development. This study was based on the US samples, while the prevalence in other countries/areas, particularly developing countries, should be further investigated.

## Conclusion

Our study highlighted that obesity and abdominal obesity remained a significant public health burden during 2001–2018 in US adults using nationally representative samples. The increasing trend remained in both males and females from 2001 to 2018. From 2001–2002 to 2017–2018, a right-shifted distribution and significant increasing trend were observed in BMI and waist circumference.

## Data availability statement

The original contributions presented in the study are included in the article/Supplementary material, further inquiries can be directed to the corresponding authors.

## Author contributions

J-YS, X-QK, and WS: conception and design. X-QK, WS, and W-JH: administrative support. H-LL: data download. J-YS, YH, Y-XM, H-LL, QQ, and CC: collection and assembly of data. J-YS, Y-XM, and H-LL: data analysis and interpretation. J-YS, W-JH, YH, QQ, CC, H-LL, X-QK, Y-XM, and WS: manuscript writing. J-YS, W-JH, YH, QQ, CC, H-LL, X-QK, Y-XM, and WS: final approval of manuscript. All authors contributed to the article and approved the submitted version.

## Funding

This study was supported by the Introduced Project of Wujiang Clinical Medical Expert Team (No. WJYJTD201704) and the Postgraduate Research and Practice Innovation Program of Jiangsu Province (SJCX21_0626).

## Conflict of interest

The authors declare that the research was conducted in the absence of any commercial or financial relationships that could be construed as a potential conflict of interest.

## Publisher's note

All claims expressed in this article are solely those of the authors and do not necessarily represent those of their affiliated organizations, or those of the publisher, the editors and the reviewers. Any product that may be evaluated in this article, or claim that may be made by its manufacturer, is not guaranteed or endorsed by the publisher.

## Abbreviations

BMI: body mass index
NHANES: National Health and Nutrition Examination Survey
OR: odds ratio
CI: confidence interval.
