# Supplementary figures and images for: Trends in general and abdominal obesity in US adults: Evidence from the National Health and Nutrition Examination Survey (2001–2018)
Source: Front Public Health. 2022 Oct 6;10:925293. doi: 10.3389/fpubh.2022.925293 (PMC9582849; doi:10.3389/fpubh.2022.925293)

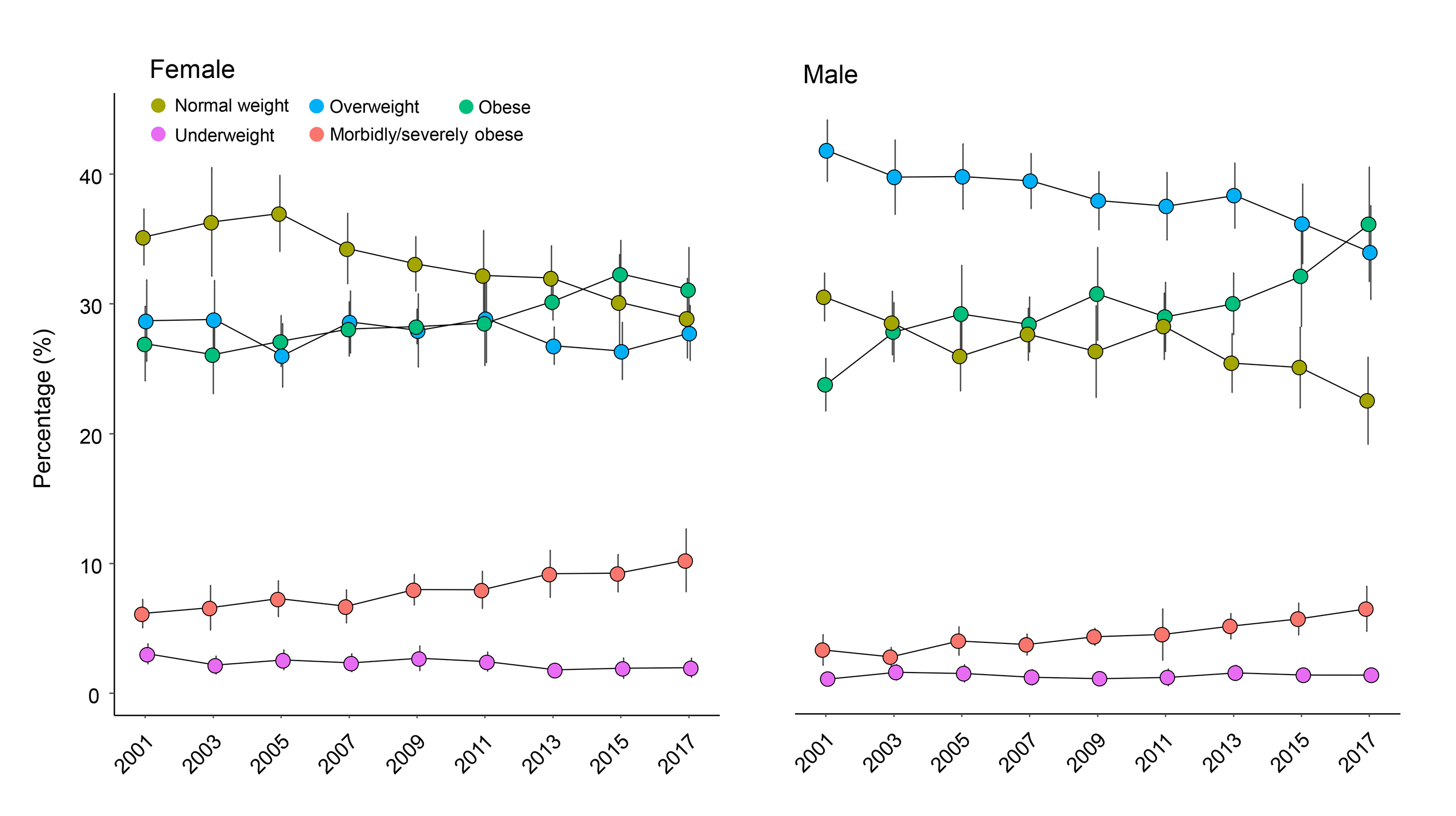

Supplement: Supplementary file 1 [file Image_1.TIF]
